# Reviewer Acknowledgment 2015

**DOI:** 10.1186/s10194-016-0595-z

**Published:** 2016-02-10

**Authors:** Valerio De Angelis

**Affiliations:** Department of Clinical and Molecular Medicine, Sapienza University of Rome, Rome, Italy

## Abstract

The quality of *The Journal of Headache and Pain* depends on the qualified and regular collaboration of renowned scientists, who devoted their time to constructively review the submitted articles. We are indebted to the following experts who reviewed papers that completed the peer-reviewing process within the year 2015.

**Fayyaz Ahmed**

United Kingdom

**Mohammed Al Jumah**

Saudi Arabia

**Lucia Albers**

Germany

**Gianni Allais**

Italy

**Fabio Antonaci**

Italy

**Roberto Arcioni**

Italy

**Sait Ashina**

United States of America

**Messoud Ashina**

Denmark

**Ilya Ayzenberg**

Germany

**Ramesh Balasubramaniam**

Australia

**Ettore Beghi**

Italy

**Massimiliano Beghi**

Italy

**Andrea Carmine Belin**

Sweden

**Robert Belvis**

Spain

**Lars Bendtsen**

Denmark

**Débora Bevilaqua-Grossi**

Brazil

**Hayrunnisa Bolay**

Turkey

**Francesco Bono**

Italy

**Marina Borro**

Italy

**David Borsook**

United States of America

**Guy Boudreau**

Canada

**Filippo Brighina**

Italy

**Oliviero Bruni**

Italy

**Rami Burstein**

United States of America

**Martin Burtscher**

Austria

**Dawn Buse**

United States of America

**Roger Cady**

United States of America

**Alessandro Capuano**

Italy

**Cinzia Cavestro**

Italy

**Sabina Cevoli**

Italy

**W. Chen**

Taiwan

**Shih-Pin Chen**

Taiwan

**Yu Chwen Chiu**

Taiwan

**I-Ching Chou**

Taiwan

**Nikolaos Christidis**

Sweden

**Carlo Cianchetti**

Italy

**Beatrice Cioni**

Italy

**Daniela Cologno**

Italy

**Gianluca Coppola**

Italy

**Cinzia Costa**

Italy

**Martina Curto**

Italy

**Valerio De Angelis**

Italy

**Willem De Hertogh**

Belgium

**Robert de Kanter**

Netherlands

**Marina de Tommaso**

Italy

**Emmanouil Dermitzakis**

Greece

**Paola Di Fiore**

Italy

**Cherubino Di Lorenzo**

Italy

**Matteo Diano**

Italy

**Christoph Dodt**

Germany

**Izabela Domitrz**

Poland

**Justin Durham**

United Kingdom

**Maria Esposito**

Italy

**Stefan Evers**

Germany

**Cesar Fernandez-de-Las**

Spain

**Denys Fontaine**

France

**F. Freitag**

United States of America

**Jong-Ling Fuh**

Taiwan

**Federica Galli**

Italy

**Charly Gaul**

Germany

**Giovanna Gentile**

Italy

**Pierangelo Geppetti**

Italy

**Maria Adele Giamberardino**

Italy

**Raquel Gil-Gouveia**

Portugal

**Peter Goadsby**

United Kingdom

**Franco Granella**

Italy

**Lyn Griffiths**

Australia

**Brian Grosberg**

United States of America

**Simona Guerzoni**

Italy

**Knut Hagen**

Norway

**Tirril Harris**

United Kingdom

**Stavros Hatzopoulos**

Italy

**Tone Heinskou**

Denmark

**Philip Holland**

United Kingdom

**Christopher Holton**

United Kingdom

**Anders Hougaard**

Denmark

**Joyce Hurley**

United States of America

**Marco Innamorati**

Italy

**Ken Iwata**

Japan

**Pernilla Jonsson**

Sweden

**Tim Juergens**

Germany

**Marielle Kabbouche**

United States of America

**Jayantee Kalita**

India

**D. G.A. Kasteleijn-Nolst Trenite**

Netherlands

**Zaza Katsarava**

Germany

**Espen Saxhaug Kristoffersen**

Norway

**Giorgio Lambru**

Italy

**Michel Lantéri-Minet**

France

**Carolina Lemos**

Portugal

**Mattias Linde**

Norway

**Christofer Lundqvist**

Norway

**Ferdinando Maggioni**

Italy

**Donna Maier**

United States of America

**Michael Marmura**

United States of America

**Paolo Martelletti**

Italy

**Alexander Mauskop**

United States of America

**Arne May**

Germany

**Francesco Saverio Mennini**

Italy

**Saras Menon**

Australia

**Marco Mercieri**

Italy

**Robert Merrill**

United States of America

**Matteo Morotti**

Italy

**Andrea Negro**

Italy

**Bao Nguyen**

Australia

**Mark Obermann**

Germany

**Petter Moe Omland**

Norway

**Serena Orr**

Canada

**Tomris Ozben**

Turkey

**Raffaele Palmirotta**

Italy

**Stefano Palmisani**

Italy

**Juan Pareja**

Spain

**Pasquale Parisi**

Italy

**Julio Pascual**

Spain

**Maurizio Pompili**

Italy

**Luiz Paulo Queiroz**

Brazil

**Alberto Raggi**

Italy

**Ana Recober**

United States of America

**Uwe Reuter**

Germany

**Michael Bjørn Russell**

Norway

**Antonio Russo**

Italy

**Angelo Russo**

Italy

**Simona Sacco**

Italy

**Pradeep Sahota**

United States of America

**Paola Sarchielli**

Italy

**Joerg Scheidt**

Germany

**Erich Schmutzhard**

Austria

**Jean Schoenen**

Belgium

**Markus Schuerks**

Germany

**Laura Schulte**

Germany

**Henrik Schytz**

Denmark

**Macit Selekler**

Turkey

**Gianluca Serafini**

Italy

**Akiko Shimada**

Denmark

**Stephen Silberstein**

United States of America

**Aksel Siva**

Turkey

**Cathy Stark**

Australia

**Amaal Starling**

United States of America

**Timothy Steiner**

Norway

**Lars Jacob Stovner**

Norway

**Andreas Straube**

Germany

**Shani Stuart**

Australia

**Sergey Sudakov**

Russian Federation

**Keisuke Suzuki**

Japan

**Claudio Tana**

Italy

**Cristina Tassorelli**

Italy

**Salvatore Terrazzino**

Italy

**Alessandro Tessitore**

Italy

**Peer Tfelt-Hansen**

Denmark

**Bernd Tomandl**

Germany

**Paola Torelli**

Italy

**Q. Tran**

Vietnam

**Erling Tronvik**

Norway

**Marco Trucco**

Italy

**Andrea Truini**

Italy

**Massimiliano Valeriani**

Italy

**László Vécsei**

Hungary

**Alberto Verrotti**

Italy

**Roxanne Walder**

United States of America

**Swen Widmalm**

United States of America

**Cicek Wöber-Bingöl**

Germany

**Jinglong Wu**

Japan

**Wang Yuan**

China

**Joanna Zakrzewska**

United Kingdom

